# Molecular subtypes predict second breast events of ductal carcinoma in situ after breast‐conserving surgery

**DOI:** 10.1002/cam4.4651

**Published:** 2022-05-22

**Authors:** Yilan Yang, Xu Zhao, Xuanyi Wang, Kairui Jin, Jurui Luo, Zhaozhi Yang, Xin Mei, Jinli Ma, Zhimin Shao, Zhen Zhang, Xingxing Chen, Xiaomao Guo, Xiaoli Yu

**Affiliations:** ^1^ Department of Radiation Oncology Fudan University Shanghai Cancer Center Shanghai China; ^2^ Department of Oncology, Shanghai Medical College Fudan University Shanghai China; ^3^ Shanghai Key Laboratory of Radiation Oncology Shanghai China; ^4^ Department of Breast Surgery Fudan University Shanghai Cancer Center Shanghai China

**Keywords:** DCIS, HER2 overexpression, molecular subtypes, second breast events

## Abstract

**Purpose:**

Currently, the prognostic value of molecular subtypes in ductal carcinoma in situ (DCIS) remains unclear. In this study, we explored whether molecular subtypes could predict second breast events (SBEs) in patients after breast‐conserving surgery (BCS).

**Methods:**

From January 2008 to December 2016, 291 DCIS patients treated with BCS were retrospectively analyzed. Patients were classified into four molecular subtypes: luminal A, luminal B, human epidermal growth factor receptor 2 (HER2) overexpression, and triple‐negative breast cancer (TNBC). The SBE incidence was calculated by the competing risk model and compared by Gray's test. The disease‐free survival rates were estimated by the Kaplan–Meier method and compared by the log‐rank test. Prognostic factors were evaluated by univariate and multivariate COX proportional hazards regression model.

**Results:**

With a median follow‐up of 66 months, 12 SBEs were identified. The 5‐year overall SBE incidence of luminal A, luminal B, HER2 overexpression, and TNBC was 2.18%, 4.25%, 15.15%, and 0.00%, respectively. In the univariate analysis, the HER2 overexpression subtype was the predictor of overall (*p* = 0.005), in situ (*p* = 0.004), and ipsilateral SBEs (*p* = 0.008). Patients with endocrine therapy were less likely to develop in situ SBEs (*p* = 0.039). Additionally, patients with closed (<2 mm) or involved margins were related to a higher risk of contralateral SBEs (*p* = 0.029). In the multivariate analysis, the HER2 overexpression subtype remained of prognostic values for overall (*p* = 0.006), in situ (*p* = 0.029), and ipsilateral SBEs (*p* = 0.012).

**Conclusions:**

The molecular subtype, especially the HER2 overexpression subtype, was the independent prognostic factor for DCIS patients who underwent BCS.

## 1 INTRODUCTION

Ductal carcinoma in situ (DCIS) is non‐invasive breast cancer, which refers to the neoplastic lesions confined to the breast ducts with no evidence of invasion into adjacent tissues.^1^ With the popularity of mammography screening, the incidence of DCIS has dramatically increased in the last few decades.^2^ Currently, DCIS comprises approximately 20% of newly diagnosed breast malignancies in China.^3^, ^4^ Although DCIS is not a life‐threatening disease, the overall recurrence, especially invasive recurrence, is still a major concern as invasive recurrence has the potential of metastasis and increases the mortality risk of DCIS.^5^ Therefore, predicting the risk factors of DCIS recurrence and avoiding overtreatment could be the priorities of the treatment strategy.

Investigators have made great efforts to reduce local recurrence. Five randomized clinical trials (RCTs; NSABP B‐17,^6^ EORCT 10853,^7^ SweDCIS,^8^ UK/ANZ,^9^ RTOG 9804^10^) demonstrated that radiotherapy after breast‐conserving surgery (BCS) reduced the risk of local recurrence by approximately 50%. Consequently, the standard treatment for most DCIS patients is BCS followed by postoperative radiotherapy.^11^ As to the endocrine therapy, NSABP B‐24 trial indicated that the use of tamoxifen reduced 32% of invasive ipsilateral recurrence compared with radiation alone.^11^ In UK/ANZ DCIS trial, tamoxifen lowered the incidence of recurrent ipsilateral DCIS and contralateral breast events.^9^ However, neither of the trials disclosed the hormone receptor (HR) status. Currently, hormone therapy remains an option for DCIS patients. It is noteworthy that a majority of DCIS clinical trials mainly focus on ipsilateral recurrence. A meta‐analysis of Early Breast Cancer Trialists' Collaborative Group (EBCTCG)^12^ revealed that contralateral breast events (CBEs) accounted for almost one‐third of all second breast events (SBEs), suggesting that CBEs were also one of the important DCIS failure events. Thereby, our retrospective study intended to explore the risk factors of all SBEs.

Previous investigations demonstrated that DCIS recurrence was significantly influenced by age at diagnosis, surgical margin status, family history of breast cancer, and so on.^13^, ^14^, ^15^, ^16^ The University of Southern California/Van Nuys Prognostic Index (USC/VNPI), an early established model to predict local recurrence, integrated five prognostic factors and built a scoring system with corresponding treatment recommendations.^15^ Presently, the most widely used predictive tool is the Oncotype DX DCIS Recurrence Score (DCIS Score).^17^ It was the first multigene assay that estimated 10‐year local recurrence and served as a clinical decision‐making tool. Apart from the predictive models, biomarkers such as estrogen receptor (ER) and human epidermal growth factor receptor 2 (HER2) expression also identify patients at high risk of recurrence.^14^ The COBCG‐01 study^18^ demonstrated that positive ER status was related to reduced local recurrence (unadjusted model: HR, 0.32; 95% CI, 0.17–0.60; *p* = 0.0001; adjusted model: HR, 0.35; 95% CI, 0.13–0.98; *p* = 0.045) in 1072 DCIS patients who received BCS plus adjuvant radiotherapy. Another small sample retrospective study^19^ revealed that HER2 overexpression DCIS patients were more likely to experience local recurrence (HR, 1.98; 95% CI, 1.11–3.53; *p* = 0.02). Moreover, molecular subtypes based on the ER, progesterone receptor (PR), and HER2 status have provided a quite definite prognostic value in invasive breast cancer.^2^ However, the prognostic significance of molecular subtypes in DCIS remains unclear. Therefore, we assessed whether molecular subtypes were associated with SBEs in DCIS patients treated with BCS in this study, hoping to stratify high‐risk patients and optimize clinical decision‐making.

## 2 METHODS

### 2.1 Patients and treatments

The study was approved by the Ethics Committee Board of Fudan University Shanghai Cancer Center. From January 2008 to December 2016, 291 patients who received BCS and were pathologically confirmed with primary DCIS without microinvasion in our center were enrolled in this retrospective study. Patients with microinvasion, Paget's disease, concurrent invasive carcinoma, neoadjuvant therapy, history of previous malignancies, less than 6 months of follow‐up, and incomplete immunohistochemical (IHC) information of ER, PR, and HER2 were excluded. Age stratification was based on the USC/VNPI.^15^ Tumor size stratification was based on the RTOG 9804 trial.^10^ According to the Society of Surgical Oncology–American Society for Radiation Oncology–American Society of Clinical Oncology (SSO‐ASTRO‐ASCO) consensus guideline,^20^ the margins of at least 2 mm were considered negative. Postoperative radiotherapy and hormone therapy were considered based on the patient's histopathological status. Radiotherapy schedules included conventional 50Gy in 25 fractions to the whole breast without tumor bed boost, 60Gy in 30 fractions to the whole breast with tumor bed boost, hypofractionated 40.05Gy in 15 fractions to the whole breast, and 39.9Gy in 15 fractions or 38.5Gy in 10 fractions to the partial breast. For hormone therapy, tamoxifen or aromatase inhibitor was delivered to patients at the discretion of the physicians.

### 2.2 Molecular subtypes

We defined the molecular subtypes of DCIS based on histopathological features. The important histopathological features, in terms of ER, PR, and HER2 status, were assessed by IHC staining. The cut‐off points for ER and PR were both 5%.^21^ HER2 IHC score 0 to 1+ was defined as HER2‐negative. HER2 IHC 3+ was determined as HER2‐positive. The HER2 status of IHC 2+ patients depended on the fluorescence in situ hybridization (FISH) results. Since the role of HER2 status in DCIS treatment is still unclear, only two patients with unknown HER2 status (IHC 2+) underwent FISH test. Patients who were HER2 IHC 2+ without FISH detection were excluded from the analysis. Thereby, the molecular subtypes were defined as follows: (1) luminal A: ER and/or PR positive, HER2‐negative; (2) luminal B: ER and/or PR positive, HER2‐positive; (3) HER2 overexpression: ER‐negative, PR‐negative, and HER2‐positive; (4) triple‐negative breast cancer (TNBC): ER‐negative, PR‐negative, and HER2‐negative.

### 2.3 Follow‐up and outcomes

All patients were followed up every 3 months during the first 2 years, every 6 months in the next 3 years, and annually after that. During the follow‐up period, patients underwent physical examination, blood tests, chest CT, and breast imaging inspections (mammography, breast ultrasound, and MRI). The primary endpoint was disease‐free survival (DFS), which was defined as patients surviving without any breast cancer events, including local failure, contralateral breast cancer, and distant metastasis. The secondary endpoints were breast cancer‐specific survival (BCSS) and SBEs. BCSS was defined as the time from diagnosis to death from breast cancer. SBEs were defined as any treatment failure event, including secondary breast cancer, chest wall recurrence, lymph node metastasis, and distant metastasis. All SBEs in our study were secondary DCIS or invasive ductal carcinoma (IDC). There was no chest wall recurrence, lymph node, or distant metastasis observed during the follow‐up period. Here, we classified SBEs in two ways: (1) invasive SBEs and in situ SBEs and (2) ipsilateral SBEs and contralateral SBEs.

### 2.4 Statistical analysis

The patient characteristics between groups were compared by the Pearson's chi‐square test. The distribution of the variable “Follow‐up time” was analyzed by the Kruskal–Wallis test. The DFS rates were calculated by the Kaplan–Meier method and compared by the log‐rank test. The cumulative incidence of SBEs was estimated by the competing risk model and compared by Gray's test.^22^ Univariate and multivariate analyses were performed by the COX proportional hazards regression model. Since all SBEs were detected by clinical symptoms, the variable “Mode of detection” was excluded in the univariate analysis. The cases with unknown tumor size, nuclear grade, and Ki‐67 status were also excluded in the univariate analysis. Variables with a *p* value <0.1 in the univariate analysis were included in the multivariate analysis. The *p* value <0.05 was considered statistically significant. The plots were made by R packages “survminer,” “survival,” and “cmprsk.” The statistical analysis was performed by SPSS version 26.0 (SPSS) and R software version 4.1.1.

## 3 RESULTS

### 3.1 Patient characteristics

According to the inclusion and exclusion criteria of the study, 291 primary DCIS patients who underwent BCS in our center were retrospectively analyzed. The median follow‐up time for the entire cohort was 66 months (range, 6–147 months; 25th to 75th percentile, 52–89 months). The 5‐year BCSS rate was 100%. The clinical characteristics of all patients are summarized in Table 1. Based on the IHC results and definition of molecular subtypes in this study, 152 patients were luminal A (52.2%), 90 patients were luminal B (30.9%), 36 patients were HER2 overexpression (12.4%), and 13 patients were TNBC (4.5%). Although no statistical significance was reached, tumor size distribution was uneven across the molecular subtypes (*p* = 0.059). The proportion of tumor size larger than 2.5 cm in luminal A, luminal B, HER2 overexpression, and TNBC was 4.6%, 6.7%, 16.7%, and 0%, respectively. The distribution of nuclear grade also varied significantly between the four molecular subtypes (*p* < 0.001). The proportion of patients with high grade in the HER2 overexpression subgroup was 61.1%, which was much higher than the other subgroups (luminal A, 5.3%; luminal B, 28.9%; TNBC, 46.2%). After BCS or re‐excisions (due to positive margins), 283 patients (97.3%) reached clear margins. 73.2% of patients received radiotherapy, including conventional radiotherapy, hypofractionated radiotherapy, and accelerated partial breast irradiation. 79.7% of patients received endocrine therapy with tamoxifen or aromatase inhibitors.

**TABLE 1:** Clinical characteristics by molecular subtype

Characteristic	Total (*n* = 291, 100%)	Luminal A (*n* = 152, 52.2%)	Luminal B (*n* = 90, 30.9%)	HER2 overexpression (*n* = 36, 12.4%)	TNBC (*n* = 13, 4.5%)	*p* value
	No. (%)	No. (%)	No. (%)	No. (%)	No. (%)	
Follow‐up time, months						
Median	66	67	63	69	63	0.388
25th to 75th percentile	52–89	56–97	51–82	48–88	43–85	
Age, years						
≤40	62 (21.3)	33 (21.7)	22 (24.4)	6 (16.7)	1 (7.7)	0.600
41–60	189 (64.9)	98 (64.5)	59 (65.6)	23 (63.9)	9 (69.2)	
>60	40 (13.7)	21 (13.8)	9 (10.0)	7 (19.4)	3 (23.1)	
Menopausal status						
Pre‐ or perimenopausal	181 (62.2)	102 (67.1)	50 (55.6)	23 (63.9)	6 (46.2)	0.194
Postmenopausal	110 (37.8)	50 (32.9)	40 (44.4)	13 (36.1)	7 (53.8)	
Laterality						
Left	158 (54.3)	87 (57.2)	45 (50.0)	18 (50.0)	8 (61.5)	0.628
Right	133 (45.7)	65 (42.8)	45 (50.0)	18 (50.0)	5 (38.5)	
Family history of malignant tumors						
No	200 (68.7)	108 (71.1)	57 (63.3)	23 (63.9)	12 (92.3)	0.148
Yes	91 (31.3)	44 (28.9)	33 (36.7)	13 (36.1)	1 (7.7)	
Mode of detection						
Clinical symptoms	196 (67.4)	107 (70.4)	53 (58.9)	27 (75.0)	9 (69.2)	0.208
Screen detected	95 (32.6)	45 (29.6)	37 (41.1)	9 (25.0)	4 (30.8)	
Tumor size, cm						
≤2.5	261 (89.7)	139 (91.4)	79 (87.8)	30 (83.3)	13 (100.0)	0.059
>2.5	19 (6.5)	7 (4.6)	6 (6.7)	6 (16.7)	0 (0.0)	
Unknown	11 (3.8)	6 (3.9)	5 (5.6)	0 (0.0)	0 (0.0)	
Nuclear grade						
Low	87 (29.9)	73 (48.0)	13 (14.4)	0 (0.0)	1 (7.7)	<0.001***
Intermediate	134 (46.0)	65 (42.8)	49 (54.4)	14 (38.9)	6 (46.2)	
High	62 (21.3)	8 (5.3)	26 (28.9)	22 (61.1)	6 (46.2)	
Unknown	8 (2.7)	6 (3.9)	2 (2.2)	0 (0.0)	0 (0.0)	
Margins						
Free (≥2 mm)	283 (97.3)	145 (95.4)	90 (100.0)	35 (97.2)	13 (100.0)	0.182
Close (<2 mm) or involved	8 (2.7)	7 (4.6)	0 (0.0)	1 (2.8)	0 (0.0)	
Radiotherapy						
Yes	213 (73.2)	103 (67.8)	70 (77.8)	29 (80.6)	11 (84.6)	0.164
No	78 (26.8)	49 (32.2)	20 (22.2)	7 (19.4)	2 (15.4)	
Hormone therapy						
Yes	232 (79.7)	140 (92.1)	80 (88.9)	7 (19.4)	5 (38.5)	<0.001***
No	59 (20.3)	12 (7.9)	10 (11.1)	29 (80.6)	8 (61.5)	

Abbreviations: HER2, human epidermal growth factor receptor 2; TNBC, triple‐negative breast cancer.

*** *p* < 0.001.

### 3.2 Patterns of SBEs

**FIGURE 1:**
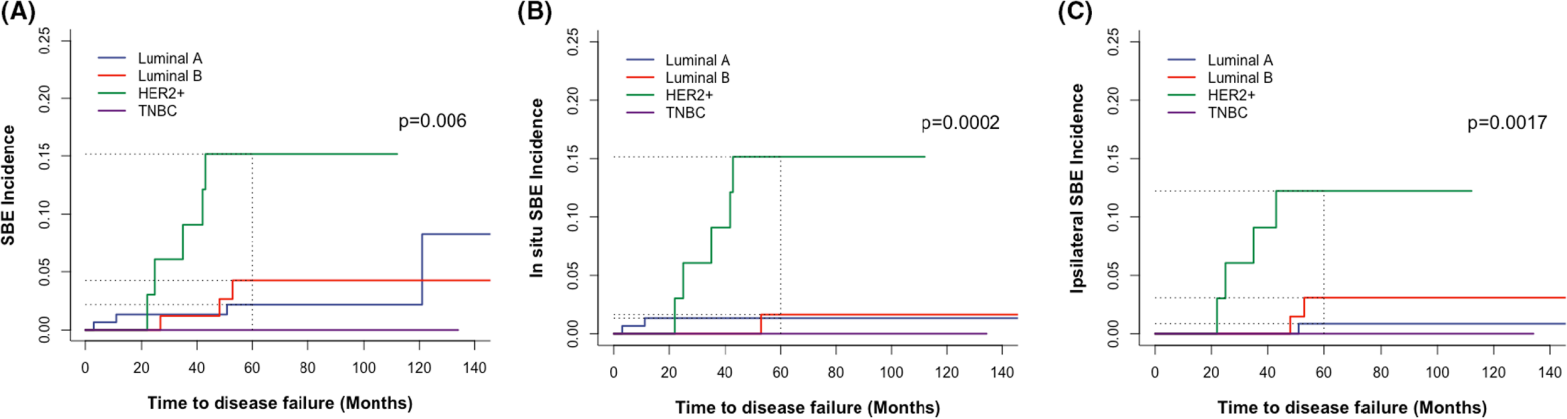
Cumulative incidence of (A) overall SBEs, (B) in situ SBEs, and (C) ipsilateral SBEs by molecular subtypes. HER2+, HER2 overexpression; TNBC, triple‐negative breast cancer

In the entire cohort, a total of 12 patients (12/291, 4.1%) developed overall SBEs, of which 4 patients (33.3%) were luminal A, 3 patients (25.0%) were luminal B, and the rest 5 patients (41.7%) were HER2 overexpression. During the follow‐up period, there were no recurrences of the TNBC subtype (Table 2). Also, no distant metastasis occurred in patients with SBEs. We analyzed SBEs in two ways: (1) based on the infiltration pattern, there were eight in situ SBEs (66.7%) and four invasive SBEs (33.3%) and (2) According to the recurrence laterality, there were seven ipsilateral SBEs (58.3%) and five contralateral SBEs (41.7%) (Table 2). Since the follow‐up time range of the HER2 overexpression subtype was 11–112 months, we only presented the SBE incidence by molecular subtypes in the first 100 months of follow‐up in Table 3. The 5‐year overall SBE cumulative incidence of luminal A, luminal B, HER2 overexpression, and TNBC was 2.18%, 4.25%, 15.15%, and 0.00%, respectively (*p* = 0.006, Figure 1A, and Table 3). Surprisingly, all HER2 overexpression subtype SBEs occurred in the first 50 months of follow‐up. However, the luminal A and luminal B SBEs occurred relatively late, more than half of which occurred after 40 months of follow‐up. In situ and ipsilateral SBE incidence data also reached similar conclusions: the HER2 overexpression subtype exhibited the highest incidence of SBEs, the luminal A subtype showed the lowest incidence of SBEs, and the SBE incidence of luminal B subtype was in‐between luminal A and HER2 overexpression. The *P* value of in situ and ipsilateral SBE incidence between the four subtypes was 0.0002 and 0.0017, respectively (Figure 1B,C, and Table 3).

**TABLE 2:** Patterns of SBEs

SBEs	Total	Luminal A	Luminal B	HER2 overexpression	TNBC
	No. (%)	No. (%)	No. (%)	No. (%)	No. (%)
Overall SBEs	12 (100.0)	4 (33.3)	3 (25.0)	5 (41.7)	0 (0.0)
Classified by infiltration pattern					
In situ SBEs	8 (66.7)	2 (16.7)	1 (8.3)	5 (41.7)	0 (0.0)
Invasive SBEs	4 (33.3)	2 (16.7)	2 (16.7)	0 (0.0)	0 (0.0)
Classified by laterality					
Ipsilateral SBEs	7 (58.3)	1 (8.3)	2 (16.7)	4 (33.3)	0 (0.0)
Contralateral SBEs	5 (41.7)	3 (25.0)	1 (8.3)	1 (8.3)	0 (0.0)

Abbreviations: HER2, human epidermal growth factor receptor 2; SBEs, second breast events; TNBC, triple‐negative breast cancer.

**TABLE 3:** Cumulative incidence of SBEs by molecular subtypes after every 20‐months follow‐up

SBEs	Time (months)	Luminal A	Luminal B	HER2 overexpression	TNBC
Overall SBEs	20	1.33%	0.00%	0.00%	0.00%
	40	1.33%	1.20%	9.09%	0.00%
	60	2.18%	4.25%	15.15%	0.00%
	80	2.18%	4.25%	15.15%	0.00%
	100	2.18%	4.25%	15.15%	0.00%
In situ SBEs	20	1.33%	0.00%	0.00%	0.00%
	40	1.33%	0.00%	9.09%	0.00%
	60	1.33%	1.64%	15.15%	0.00%
	80	1.33%	1.64%	15.15%	0.00%
	100	1.33%	1.64%	15.15%	0.00%
Ipsilateral SBEs	20	0.00%	0.00%	0.00%	0.00%
	40	0.00%	0.00%	9.09%	0.00%
	60	0.85%	3.09%	12.23%	0.00%
	80	0.85%	3.09%	12.23%	0.00%
	100	0.85%	3.09%	12.23%	0.00%

Abbreviations: HER2, human epidermal growth factor receptor 2; SBEs, second breast events; TNBC, triple‐negative breast cancer.

### 3.3 Prognostic factors of SBEs

**FIGURE 2:**
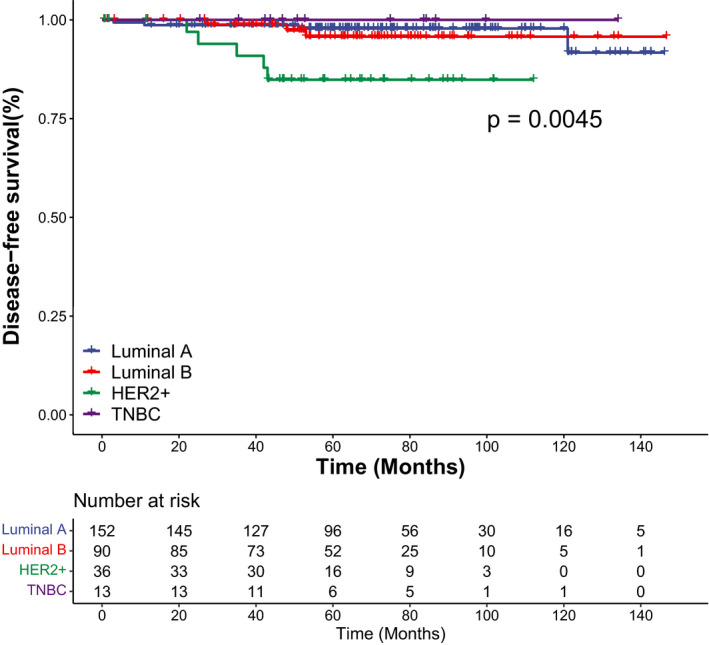
Kaplan–Meier curves of disease‐free survival by molecular subtypes. HER2+, HER2 overexpression; TNBC, triple‐negative breast cancer

**FIGURE 3:**
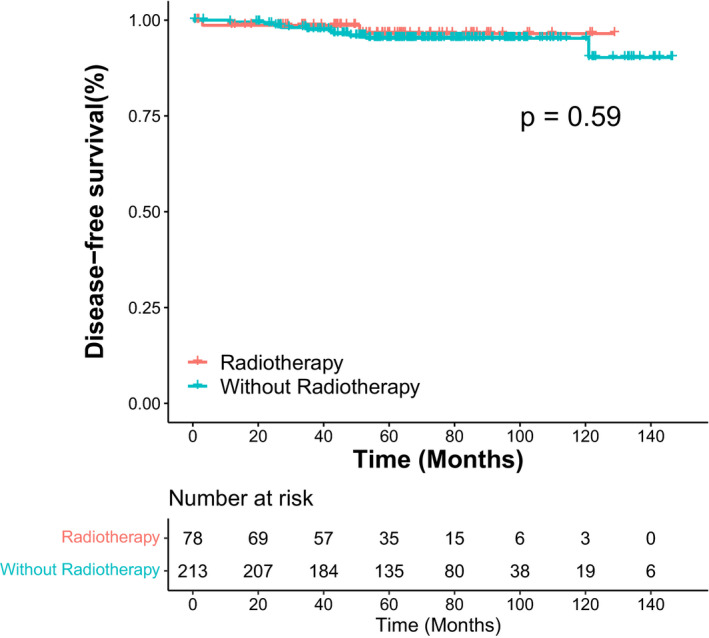
Kaplan–Meier curves of disease‐free survival by radiotherapy

In univariate COX analysis, molecular subtypes predicted overall SBEs. Compared with the luminal A subtype, the HER2 overexpression subtype presented the highest risk of overall SBEs (HR, 7.2; 95% CI, 1.8–28.1; *p* = 0.005). Age, menopausal status, laterality, family history of malignant tumors, tumor size, nuclear grade, margin status, radiotherapy, hormone therapy, and Ki‐67 status failed to predict overall SBEs (Table 4). We also analyzed whether these prognostic factors were associated with the SBE subgroups (Table 5 and Table 6). For in situ SBEs, the HER2 overexpression subtype (HR, 11.4; 95% CI, 2.2–59.0; *p* = 0.004) remained the independent prognostic factor. Moreover, patients with hormone therapy were less likely to experience in situ SBEs (HR, 0.2; 95% CI, 0.1–0.9; *p* = 0.039). We did not identify any independent prognostic factor associated with invasive SBEs. For ipsilateral SBEs, the HER2 overexpression subtype still exhibited the predictive value (HR, 19.9; 95% CI, 2.2–179.0; *p* = 0.008). Interestingly, patients with closed (<2 mm) or involved margins were more likely to develop contralateral SBEs (HR, 12.5; 95% CI, 1.3–120.2; *p* = 0.029). In multivariate COX analysis, the HER2 overexpression subtype still predicted overall SBEs (HR, 7.0; 95% CI, 1.8–27.8; *p* = 0.006), in situ SBEs (HR, 11.1; 95% CI, 1.3–96.7; *p* = 0.029), and ipsilateral SBEs (HR, 26.0; 95% CI, 2.0–332.9; *p* = 0.012; Table 7). The Kaplan–Meier survival curves of DFS by molecular subtypes and radiotherapy are displayed in Figures 2 and 3.

**TABLE 4:** Univariate analysis of risk factors for overall SBEs

Variable	Overall SBEs (*n* = 12, 100%)		
	HR	95% CI	*p* Value
Age, years (vs ≤40)			
41–60	0.5	0.1–1.7	0.259
>60	0.7	0.1–4.0	0.728
Menopausal status (postmenopausal vs pre‐ or perimenopausal)	0.5	0.1–1.9	0.335
Laterality (right vs left)	1.2	0.4–3.7	0.768
Family history of malignant tumors (Yes vs No)	2.7	0.9–8.6	0.089
Tumor size, cm (>2.5 vs ≤2.5)	2.6	0.6–12.0	0.215
Nuclear grade (vs low)			
Intermediate	2.5	0.5–12.0	0.237
High	1.5	0.2–11.0	0.666
Margins (closed [<2 mm] or involved vs free [≥2 mm])	3.9	0.5–30.2	0.197
Radiotherapy (yes vs no)	1.5	0.3–7.0	0.589
Hormone therapy (yes vs no)	0.4	0.1–1.4	0.169
Ki‐67 (high [≥15%] vs low [<15%])	0.9	0.8–1.1	0.303
Molecular subtype (vs luminal A)			
Luminal B	1.4	0.3–6.4	0.648
HER2 overexpression	7.2	1.8–28.1	0.005**
TNBC	No event	—	—

Abbreviations: CI, confidence interval; HER2, human epidermal growth factor receptor 2; HR, hazard ratio; SBEs, second breast events; TNBC, triple‐negative breast cancer

** *p* < 0.01.

**TABLE 5:** Univariate analysis of risk factors for in situ and invasive SBEs

Variable	In situ SBEs (*n* = 8, 66.7%)			Invasive SBEs (*n* = 4, 33.3%)		
	HR	95% CI	*p* value	HR	95% CI	*p* value
Age, years (vs ≤40)						
41–60	0.6	0.1–3.5	0.617	0.3	0.04–2.3	0.251
>60	1.4	0.2–10.2	0.714	No event	—	—
Menopausal status (postmenopausal vs pre‐ or perimenopausal)	0.9	0.2–3.9	0.935	No event of postmenopause		
Laterality (right vs left)	1.2	0.3–4.8	0.786	1.1	0.2–8.1	0.899
Family history of malignant tumors (yes vs no)	3.4	0.8–14.2	0.095	1.8	0.2–12.7	0.573
Tumor size, cm (>2.5 vs ≤2.5)	4.5	0.9–22.2	0.066	No event of tumor size >2.5 cm		
Nuclear grade (vs low)						
Intermediate	3.3	0.4–28.2	0.276	1.8	0.2–17.3	0.613
High	3.0	0.3–32.6	0.376	No event	—	—
Margins (closed [<2 mm] or involved vs free [≥2 mm])	5.4	0.7–43.7	0.116	No event of closed or involved margins		
Radiotherapy (yes vs no)	2.3	0.3–18.5	0.443	0.8	0.1–7.7	0.837
Hormone therapy (yes vs no)	0.2	0.1–0.9	0.039*	No event of not receiving hormone therapy		
Ki‐67 (high [≥15%] vs low [<15%])	0.7	0.1–3.5	0.656	3.4	0.3–37.5	0.318
Molecular subtype (vs luminal A)						
Luminal B	0.9	0.1–9.6	0.912	2.0	0.3–14.6	0.487
HER2 overexpression	11.4	2.2–59.0	0.004**	No event	—	—
TNBC	No event	—	—	No event	—	—

Abbreviations: CI, confidence interval; HER2, human epidermal growth factor receptor 2; HR, hazard ratio; SBEs, second breast events; TNBC, triple‐negative breast cancer.

* *p* < 0.05

** *p* < 0.01.

**TABLE 6:** Univariate analysis of risk factors for ipsilateral and contralateral SBEs

Variable	Ipsilateral SBEs (*n* = 7, 58.3%)			Contralateral SBEs (*n* = 5, 41.7%)		
	HR	95% CI	*p* value	HR	95% CI	*p* value
Age, years (vs ≤40)						
41–60	0.7	0.1–3.6	0.635	0.3	0.04–2.2	0.239
>60	0.7	0.1–7.9	0.788	0.8	0.1–8.5	0.834
Menopausal status (postmenopausal vs pre‐ or perimenopausal)	0.6	0.1–3.2	0.566	0.4	0.05–3.6	0.420
Laterality (right vs left)	0.9	0.2–4.1	0.921	1.7	0.3–10.1	0.570
Family history of malignant tumors (yes vs no)	2.7	0.6–11.9	0.200	2.8	0.5–16.9	0.262
Tumor size, cm (>2.5 vs ≤2.5)	2.3	0.3–18.7	0.451	3.1	0.3–28.0	0.310
Nuclear grade						
Low	No event	—	—	1.0		
Intermediate	1.1	0.2–5.7	0.907	0.9	0.2–5.5	0.920
High	1.0			No event	—	—
Margins (closed [<2 mm] or involved vs free [≥2 mm])	No event of closed or involved margins			12.5	1.3–120.2	0.029*
Radiotherapy (yes vs no)	1.8	0.2–15.3	0.570	1.2	0.1–10.8	0.875
Hormone therapy (yes vs no)	0.3	0.1–1.3	0.096	0.9	0.1–8.0	0.919
Ki‐67 (high [≥15%] vs low [<15%])	1.7	0.3–8.5	0.513	0.6	0.1–5.5	0.633
Molecular subtype (vs luminal A)						
Luminal B	3.5	0.3–38.8	0.304	0.7	0.1–6.4	0.716
HER2 overexpression	19.9	2.2–179.0	0.008**	2.0	0.2–21.0	0.560
TNBC	No event	—	—	No event	—	—

Abbreviations: CI, confidence interval; HER2, human epidermal growth factor receptor 2; HR, hazard ratio; SBEs, second breast events; TNBC, triple‐negative breast cancer.

* *p* < 0.05

** *p* < 0.01.

**TABLE 7:** Multivariate analysis of risk factors for overall, in situ, and ipsilateral SBEs

Variable	Overall SBEs			In situ SBEs		Ipsilateral SBEs			
	HR	95% CI	*p* value	HR	95% CI	*p* value	HR	95% CI	*p* value
Family history of malignant tumors (yes vs no)									
	2.6	0.8–8.1	0.110	4.1	0.9–18.9	0.070	—	—	—
Tumor size, cm (>2.5 vs ≤2.5)									
	—	—	—	4.1	0.6–26.4	0.136	—	—	—
Hormone therapy (yes vs no)									
	—	—	—	1.6	0.2–12.1	0.654	1.5	0.2–10.4	0.700
Molecular subtype (vs luminal A)									
Luminal B	1.3	0.3–6.0	0.716	0.8	0.1–8.4	0.819	3.6	0.3–39.3	0.299
HER2 overexpression	7.0	1.8–27.8	0.006*	11.1	1.3–96.7	0.029*	26.0	2.0–332.9	0.012*
TNBC	No event	—	—	No event	—	—	No event	—	—

Abbreviations: CI, confidence interval; HER2, human epidermal growth factor receptor 2; HR, hazard ratio; SBEs, second breast events; TNBC, triple‐negative breast cancer.

* *p* < 0.05.

## 4 DISCUSSION

With the early screening of breast cancer, the incidence of DCIS has dramatically increased and many patients face the risk of SBEs. In addition, the prognostic value of molecular subtypes in invasive breast cancer is well addressed and definite, while its role in DCIS prognosis remains unclear. Our study described the patterns of SBEs in different molecular subtypes and demonstrated that the HER2 overexpression subtype was an independent prognostic predictor for DCIS patients who received BCS.

With a median follow‐up of 5.5 years, 12 SBEs were identified in 291 patients. The 5.5‐year ipsilateral SBE incidence, so‐called local recurrence (LR) rate, was 2.4% (7/291) in this study, which was much lower than prior prospective DCIS RCTs. The 5.2‐year LR rate was 15.4% in the SweDCIS trial^23^ and the 4.25‐year LR rate was 13.6% in the EORCT 10853 trial.^24^ The discrepancy could be explained in the following two sides. First, the two RCTs mentioned above were randomized trials to investigate the effects of radiotherapy in DCIS local control, thus only half of the patients received radiation, while in this study, 73.2% of patients received radiotherapy. Second, the margin negativity rate was much higher in this study (97.3%) compared to the SweDCIS trial (80.3%) and the EORCT 10853 trial (74.6%). Although the LR rate in our study varied a lot from the SweDCIS and EORCT 10853 trials, it was generally consistent with trials bearing modern management, such as RTOG 9804 trial. The 7‐year LR rate was 0.9% in the radiotherapy arm and 6.7% in the observation arm in the RTOG 9804 trial.^10^ Limited prognostic information was found due to the low SBE incidence in our study. We discovered three predictors in the univariate analysis, which were positive margins (*p* = 0.029), lack of hormone therapy (*p* = 0.039), and the HER2 overexpression subtype (*p* = 0.005 for overall SBEs; *p* = 0.004 for in situ SBEs; *p* = 0.008 for ipsilateral SBEs). Only the HER2 overexpression subtype remained significant (*p* = 0.006 for overall SBEs; *p* = 0.029 for in situ SBEs; *p* = 0.012 for ipsilateral SBEs) in the multivariate analysis.

Several RCTs have demonstrated that positive margins increase the risk of local recurrence in DCIS.^6^, ^13^, ^14^, ^25^ The EORTC 10853 trial^24^ reported a 1.69‐fold higher risk (HR, 1.69; 95% CI, 1.23–2.31; *p* < 0.001) of local recurrence in patients with closed (<1 mm) or involved margins than free margins after BCS. The NSABP B‐24 trial^6^ revealed that the 15‐year local recurrence rate was 17.4% in patients with involved margins, while 7.4% in patients with tumor‐free margins (HR, 2.61; 95% CI, 1.68–4.05; *p* < 0.001). However, we found that closed (<2 mm) or involved margins was an independent prognostic factor for contralateral SBEs (unadjusted HR, 12.5; 95% CI, 1.3–120.2; *p* = 0.029) instead of ipsilateral SBEs. The underlying reason may be listed below. In all patients who developed SBEs, only one patient had positive margins and experienced a contralateral SBE. There was no event of positive margins in patients who experienced ipsilateral SBEs. Therefore, we were not able to compare the effects of different margin statuses on ipsilateral SBE incidence. The low incidence of positive margins in relapsed (1/12, 8.3%) and overall population (8/291, 2.7%) could lead to biased results.

Previous RCTs^6^, ^9^, ^26^ reported that patients with endocrine therapy presented a better prognosis compared to those without endocrine therapy. According to the UK‐ANZ and NSABP B‐24 trials, tamoxifen lowered the risk of ipsilateral and contralateral breast cancer by approximately 30%–50%.^27^ Since the two RCTs started about 20 years ago, patients were enrolled with unknown HR status. One subgroup analysis based on NSABP B‐24^26^ retrospectively evaluated the HR status and proposed that postoperative tamoxifen in ER‐positive DCIS patients significantly decreased overall SBEs (HR, 0.58; 95% CI, 0.42–0.81; *p* = 0.002) and invasive SBEs (HR, 0.53; 95% CI, 0.34–0.82; *p* = 0.005). Reductions were also observed in in situ SBEs, although it was statistically nonsignificant (HR, 0.66; 95% CI, 0.39–1.12; *p* = 0.12). However, we discovered that hormone therapy reduced the in situ SBE risks (unadjusted HR, 0.2; 95% CI, 0.1–0.9; *p* = 0.039) with statistical significance. The divergence was probably due to the fact that the administration of hormone therapy was not completely based on the HR status in our study. 7.7% of luminal A patients did not receive endocrine therapy, while 22.2% of TNBC patients received endocrine therapy. Moreover, the NSABP B‐24 only utilized tamoxifen, whereas the endocrine agents in our study also included aromatase inhibitors.

The molecular subtype of HER2 overexpression was found to be a prognostic factor for overall (unadjusted HR, 7.2; 95% CI, 1.8–28.1; *p* = 0.005; adjusted HR, 7.0; 95% CI, 1.8–27.8; *p* = 0.006), in situ (unadjusted HR, 11.4; 95% CI, 2.2–59.0; *p* = 0.004; adjusted HR, 11.1; 95% CI, 1.3–96.7; *p* = 0.029), and ipsilateral SBEs (unadjusted HR, 19.9; 95% CI, 2.2–179.0; *p* = 0.008; adjusted HR, 26.0; 95% CI, 2.0–332.9; *p* = 0.012) in our study. So far, several studies^19^, ^21^, ^28^, ^29^ have supported our findings. Curigliano et al.^28^ demonstrated that HER2 overexpression significantly increased in situ breast cancer recurrence (HR, 1.59; 95% CI, 1.06–2.39; *p* = 0.01). Han et al.^19^ discovered that HER2 overexpression was more likely to experience local recurrence (HR, 1.98; 95% CI, 1.11–3.53; *p* = 0.02). Thorat et al.^29^ yielded similar conclusions that HER2 overexpression was a predictor of ipsilateral breast events (IBEs; HR, 2.29; 95% CI, 1.64–3.14; *p* < 0.0001) and in situ IBEs (HR, 2.90; 95% CI, 1.91–4.40; *p* < 0.0001). The studies mentioned above are in high agreement with our results. Furthermore, the HER2 overexpression subtype presented the highest risk of SBEs and showed the earliest recurrence. The intrinsic tumor characteristics may explain the finding. First, the proportion of patients with tumors larger than 2.5 cm were 16.7% in the HER2 overexpression subtype, which was the highest among four subtypes (luminal A, 4.6%; luminal B, 6.7%; TNBC, 0%). Another key point was that 61.1% of the HER2 overexpression tumors were nuclear high grade. However, the percentage of high‐grade tumors in luminal A, luminal B, and TNBC was 5.3%, 28.9%, and 46.2%, respectively. Cesare et al.^30^ confirmed that HER2 positive status in DCIS was associated with high nuclear grade (*p* < 0.001) and high Ki‐67 expression (*p* = 0.003). Based on these findings, the NSABP B‐43 study^31^ explored whether the use of trastuzumab in HER2‐positive DCIS patients could reduce ipsilateral recurrence. After 79.2 months of follow‐up, the radiotherapy plus trastuzumab arm achieved a modest, but statistically nonsignificant decrease of 19% in ipsilateral recurrence rate.

Another intriguing phenomenon is that no SBE occurred in the TNBC cohort, which may be partly related to the clinical characteristics of the TNBC group. In the TNBC cohort, all tumors were smaller than 2.5 cm and all patients had clear margins. On the other hand, the small sample size, the low incidence of triple‐negative DCIS, and insufficient follow‐up time made it difficult to identify SBEs. Interestingly, Williams et al.^21^ proposed that other than the HER2 overexpression subtype, luminal B and TNBC predicted overall recurrence as well (luminal A as the reference; luminal B, HR 5.14, *p* = 0.001; HER2 overexpression, HR 6.46, *p* < 0.001; TNBC, HR 3.27, *p* = 0.028). Liu et al.^32^ also found that the TNBC subtype exhibited the poorest OS (HR, 3.88; 95% CI, 1.62–9.29; *p* = 0.002). However, the median follow‐up time of the study was only 42 months, and the data was derived from the Surveillance, Epidemiology, and End Results (SEER) database, thereby lack of specific recurrence data. Moreover, Williams et al.^21^ demonstrated that the luminal A subtype was significantly related to better DFS (*p* < 0.001). A possible explanation is that the luminal A subtype showed the lowest average tumor size (*p* = 0.005), the lowest proportion of high‐grade tumors (*p* < 0.001), and the lowest average Ki‐67 expression (*p* = 0.042) in their cohort. However, the investigators did not disclose the details of postoperative adjuvant therapy including radiotherapy and endocrine therapy, which was a missing piece in their study and could potentially lead to biased results. In our cohort, we did not discover any relationship between luminal A and SBEs. This may be related to the clinical characteristics of luminal A patients. Although the luminal A subtype also exhibited the lowest percentage of high‐grade tumors (*p* < 0.001), the proportion of positive margins (luminal A, 4.6%; luminal B, 0%; HER2 overexpression, 2.8%; TNBC, 0%) and without radiotherapy (luminal A, 32.2%; luminal B, 22.2%; HER2 overexpression, 19.4%; TNBC, 15.4%) was highest among all subgroups.

Multiple RCTs^6^, ^7^, ^8^, ^9^, ^10^ have confirmed that radiotherapy significantly reduced SBEs, especially ipsilateral recurrence, in patients who underwent BCS. Therefore, radiotherapy has become part of the standard treatments for DCIS patients. In our cohort, 26.8% (78/291) of patients did not receive radiotherapy after BCS. Moreover, radiotherapy failed to predict SBEs or DFS in the univariate COX analysis (*p* = 0.589 for overall SBEs, *p* = 0.570 for ipsilateral SBEs, *p* = 0.875 for contralateral SBEs,) and survival curve analysis (*p* = 0.59). Patients without radiotherapy presented the following clinical features (Table S1): (1) 25.6% were older than 60 years; (2) 85.9% tumors were smaller than 2.5 cm; (3) 83.5% tumors were low‐to‐intermediate in nuclear grade; and (4) all patients had clear margins. Taken together, patients without radiotherapy were at low risk of recurrence. In addition, the median follow‐up time of our study was only 66 months. Therefore, we have not yet identified radiotherapy as a prognostic factor.

Regarding the conventional DCIS predictors such as tumor size and nuclear grade, there was no statistical significance of these factors on SBE risks in our analysis. Possible explanations can be listed as follows. First, the sample size of 291 patients was relatively small. Second, the number of SBEs was limited due to the low recurrence rate of DCIS in our center. Third, baseline characteristics were not well‐balanced between the four subgroups. Although the distribution of tumor size and nuclear grade was uneven, several literatures^19^, ^27^, ^29^, ^30^ have indicated that the HER2 overexpression type tended to be larger in tumor size and higher in nuclear grade.

## 5 CONCLUSION

Our findings demonstrated that the HER2 overexpression subtype was related to the increased incidence of overall, in situ, and ipsilateral SBEs. Patients with positive margins were at a higher risk of contralateral SBEs. Additionally, patients benefited from endocrine therapy with a reduced risk of in situ SBEs. Since this is a small‐sample retrospective study, large cohorts with long‐term follow‐up and complete pathological and molecular information are needed to further improve risk stratifications and provide tailored treatment for DCIS patients.

## CONFLICT OF INTEREST

The authors declare that they have no competing interests.

## AUTHOR CONTRIBUTION

Yilan Yang, Xu Zhao, Xuanyi Wang, Kairui Jin, and Jurui Luo were responsible for data collection. Yilan Yang analyzed data and drafted the first copy. Zhaozhi Yang, Xin Mei, Jinli Ma, Zhimin Shao, and Zhen Zhang prepared the figures and tables. Xiaoli Yu, Xiaomao Guo, and Xingxing Chen conceived and critically revised this work. All authors read and approved the final manuscript.

## ETHICS STATEMENT

All procedures in this retrospective study involving human participants were performed according to the ethical standards of the national research committee and the 1964 Helsinki Declaration. The study was approved by the Ethics Committee Board of Fudan University Shanghai Cancer Center.

## Supporting information


Table S1

## Data Availability

The data are available from the corresponding authors with the permission of Fudan University Shanghai Cancer Center on reasonable request.
