# Supplementary material for: Molecular subtypes predict second breast events of ductal carcinoma in situ after breast‐conserving surgery
Source: Cancer Med. 2022 May 22;11(14):2755–66. doi: 10.1002/cam4.4651 (PMC9302274; doi:10.1002/cam4.4651)
Supplement: Supplementary file 1 — Table S1 [file CAM4-11-2755-s001.docx]

**Supplementary Table 1.** Clinical characteristics by radiotherapy

| Characteristic | | Radiotherapy  (n=213, 73.2%) | Without Radiotherapy (n=78, 26.8%) | *P* Value |
| --- | --- | --- | --- | --- |
|  |  | No. (%) | No. (%) |  |
| Age, years | |  |  |  |
|  | ≤ 40 | 48(22.5) | 14(17.9) | **0.002**** |
|  | 41-60 | 145(68.1) | 44(56.4) |  |
|  | > 60 | 20(9.4) | 20(25.6) |  |
| Menopausal status | |  |  |  |
|  | Pre- or perimenopausal | 137(64.3) | 44(56.4) | 0.218 |
|  | Postmenopausal | 76(35.7) | 34(43.6) |  |
| Laterality | |  |  |  |
|  | Left | 112(52.6) | 46(59.0) | 0.332 |
|  | Right | 101(47.4) | 32(41.0) |  |
| Family history of malignant tumors | |  |  |  |
|  | No | 143(67.1) | 57(73.1) | 0.333 |
|  | Yes | 70(32.9) | 21(26.9) |  |
| Mode of detection | |  |  |  |
|  | Clinical symptoms | 135(63.4) | 61(78.2) | **0.017*** |
|  | Screen detected | 78(36.6) | 17(21.8) |  |
| Tumor Size, cm | |  |  |  |
|  | ≤ 2.5 | 194(91.1) | 67(85.9) | 0.571 |
|  | >2.5 | 13(6.1) | 6(7.7) |  |
|  | Unknown | 6(2.8) | 5(6.4) |  |
| Nuclear Grade | |  |  |  |
|  | Low | 51(23.9) | 36(46.2) | **<0.001***** |
|  | Intermediate | 105(49.3) | 29(37.2) |  |
|  | High | 53(24.9) | 9(11.5) |  |
|  | Unknown | 4(1.9) | 4(5.1) |  |
| Margins | |  |  |  |
|  | Free (≥2mm) | 205(96.2) | 78(100.0) | 0.083 |
|  | Close (<2mm) or Involved | 8(3.8) | 0(0.0) |  |
| Hormone Therapy | |  |  |  |
|  | No | 34(16.0) | 25(32.1) | **0.002**** |
|  | Yes | 179(84.0) | 53(67.9) |  |
| Molecular Subtype | |  |  |  |
|  | Luminal A | 103(48.4) | 49(62.8) | 0.164 |
|  | Luminal B | 70(32.9) | 20(25.6) |  |
|  | HER2 overexpression | 29(13.6) | 7(9.0) |  |
|  | TNBC | 11(5.2) | 2(2.6) |  |

HER2, human epidermal growth factor receptor 2; TNBC, triple-negative breast cancer; * *P*<0.05; ** *P*<0.01; *** *P*<0.001.
